# Ultra-High SNR Demodulation Method for Optical Fiber Sensors Applied in Power Transformer Partial Discharge Detection

**DOI:** 10.3390/s22082828

**Published:** 2022-04-07

**Authors:** Jixian Qiao, Weichao Zhang, Yanqi Wang, Qibing Shao, Jianlong Cai, Hong Zhao

**Affiliations:** 1 Key Laboratory of Engineering Dielectrics and Its Application, Ministry of Education, School of Electrical and Electronic Engineering, Harbin University of Science and Technology, Harbin 150080, China; 1810300010@stu.hrbust.edu.cn (J.Q.); 2020310144@stu.hrbust.edu.cn (Y.W.); 2010300016@stu.hrbust.edu.cn (Q.S.); 2 Huizhou Electric Power Survey and Design Institute Co., Ltd., Huizhou 516023, China; cjl@hzed.com.cn

**Keywords:** optical fiber sensors, DFB fiber laser, optical coupler asymmetry, improved optical demodulation

## Abstract

The demodulation method of optical fiber sensors utilized in power transformer partial discharge (PD) detection is insufficient for engineering applications. We design a distributed feedback fiber laser (DFB-FL) PD detection system with an asymmetric 3 × 3 coupler and propose an ultra-high signal-to-noise ratio (SNR) demodulation scheme by eliminating the main factors that affect the traditional method using an asymmetric 3 × 3 coupler. The power transformer PD detection results reveal that the proposed scheme is free from 3 × 3 coupler asymmetry issues, with an average SNR of 38.30 dB, which is much higher than the widely used demodulation method and the piezoelectric transducer sensor. The average SNR of the system is increased by 24.2 dB with the proposed method.

## 1. Introduction

Over the last two decades, optical fiber sensors used in power equipment to detect partial discharge (PD) acoustic emission (AE) signals have sparked a lot of attention and advancement [1,2,3,4,5,6,7,8,9,10,11,12,13]. Although there are many different optical fiber sensors for partial discharge detection, their demodulation methods may be divided into two categories depending on the ultimate demodulation objective: intensity demodulation (ID) and phase demodulation.

The ID method, which consists mainly of the passive homodyne [5,10], the active homodyne [8,13], and the quadrature interrogating method [3,4,6,7,9,11], reads the voltage change from the photodetector without additional computation except filtering. The homodyne typically uses a 3 dB coupler for fiber interferometric sensors to make the light interfere. The quadrature interrogating method is frequently employed for extrinsic Fabry–Perot and fiber Bragg grating sensors that focus on the change in light intensity induced by the spectral shift. Since the ID method has a basic method of construction and is easy to use, it has become the most widely used technique for optical fiber sensors applied in PD detection. The drift of the working point produced by noise in the surroundings and the photoelectric system, on the other hand, represents a typical problem with the ID method. Although the active homodyne can suppress the shift of the working point, the system signal-to-noise ratio (SNR) and real-time suppression impact in engineering may not be adequate. Furthermore, the real-time modulation device used in the active homodyne is costly.

Phase demodulation, in contrast to the ID method, entails demodulating phase information from the voltage obtained by the photodetector and is primarily comprised of the heterodyne [14,15,16], the homodyne using the phase generated carrier (PGC) method [17], and the passive homodyne using an ideal 3 × 3 coupler (or NPS) method [1,2,18,19]. These methods, which efficiently reduce circuit noise and are immune to fluctuations in optical power in the system, may be able to overcome the ID method’s SNR constraint. Because of its extensive dynamic measuring range and meager cost, the NPS may be the sole method adequate for detecting PD signals [2,20]. However, the NPS method is based on the output of an ideal coupler with exact symmetry, which is difficult to achieve in real-world manufacturing and applications.

Improved NPS (INPS) has been the subject of some studies [19,21]. Ref. [19] demands careful calculation, and circuit parameter debugging before the detection, as failure to do so may induce operation difficulty. Furthermore, this approach may not wholly reduce amplitude noise that varies with time. The appropriate real-time parameters in [21] were only gathered before each detection, which increases the application’s instability and makes it unclear whether it can detect signals over 20 kHz.

In this paper, we design a partial discharge detection system utilizing a cheaper asymmetrical 3 × 3 coupler and a distributed feedback fiber laser (DFB-FL) as the sensor. We also study most of the parameters that influence the NPS in the proposed system and then offer a simple operation demodulation scheme with ultra-high SNR that can be applied to any optical fiber sensors using the Michelson fiber interferometer.

## 2. DFB-FL Partial Discharge Detection System

**Figure 1:**
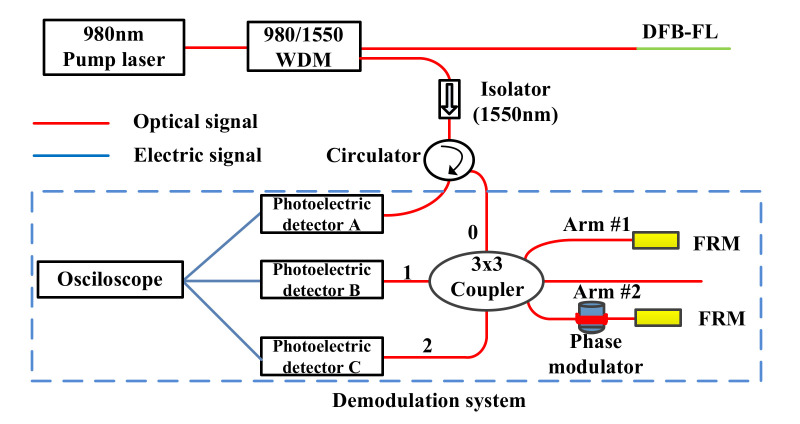
DFB-FL PD detection system (The ports of the 3 × 3 coupler are number annotated for subsequent algorithm description.).

Figure 1 depicts the DFB-FL-based partial discharge detection system. This system, which uses the ID method, can successfully identify PD signals in a liquid–solid dielectric [13], but the system’s SNR is insufficient due to working point drift and optical noise. Rayleigh backscatter is eliminated using a 1550 nm optical isolator [22], and polarization fading is suppressed using two Faraday rotating mirrors (FRM) [23]. To ensure high-frequency responsiveness and demodulation sensitivity, the length of Arm #2 is set to 50 m [24]. For the following improved NPS method, a phase modulator is embedded in Arm #2.

The relaxation oscillation center frequency of the 980 nm pump laser is 24 kHz, and this frequency in the DFB-FL output is about 600 kHz. Furthermore, the operational transformer’s Barkhausen effect noise [25] has a frequency of 50–60 kHz. To avoid the noise bands above and to remain in the PD detecting frequency band (20–500 kHz), the system detection frequency range is 60–300 kHz.

## 3. Improved NPS Method

The DC component elimination algorithm, the differential cross-multiplication (DCM) algorithm, and the automated gain control circuitry (AGC) algorithm are all part of the NPS method [18]. The processes above make the NPS method is simple to acquire phase information Φ(*t*). However, the NPS demands that all system components be ideal, which is hard to achieve in real-world PD detection, particularly in engineering applications. This means that the NPS is invalidated when an asymmetric coupler is used. The effect of an asymmetric coupler on the NPS method can be found in Ref. [19].

Other factors also impact the NPS in PD detection: Interferences from the surroundings cause the optical phase to vary by altering the DFB-FL’s central wavelength due to the DFB-FL’s superior low-frequency vibration response qualities. The DFB-FL output power fluctuation modifies output amplitude by changing the interference light intensity. The refractivity asymmetry of transmission fibers causes polarization fading in signal transmission, which impacts the output amplitude change. When light enters the photodetector, the photodetector’s gain coefficient fluctuation and inconsistency still influence the output amplitude. In other words, the asymmetric coupler introduces a phase deviation angle. The optical phase change introduces phase noise into the output. The output amplitude is no longer a constant but a time-dependent function resulting from the amplitude change. As a result, the PD detection system’s outputs employing an asymmetric 3 × 3 coupler are:

(1) $$U_{i}(t)=D_{i}(t)+A_{i}(t)\cos[\Phi (t)+\sigma (t)-(i-1)(\frac{2\pi }{3}+\theta_{i})]$$

where *i* = (0,1,2) represents the three output ports in Figure 1. *D_i_*(*t*) and *A_i_*(*t*) are the DC/AC amplitude. *θ_i_* is the asymmetric 3 × 3 coupler deviation angle. *σ*(*t*) is the phase change caused by environmental low-frequency vibration and can be expressed as *σ*(*t*) = *B*cos(*ωt*), where *B* and *ω* are the amplitude/frequency of the *σ*(*t*), respectively.

(2) $$\Phi (t)=\frac{-4\pi nL}{\lambda_{0}^{2}}\Delta \lambda (t)$$

is the partial discharge causes phase change, which is crucial information to our concern, *λ*_0_ is the steady-state wavelength, and the sensor output wavelength shift is Δ*λ*(*t*). *L* is the length of Arm#1, and *n* is the fiber refractive index. According to Equation (2), the NPS method or improved NPS scheme can be used for any optical fiber sensors using the Michelson fiber interferometer.

The improved NPS (INPS) method in [19] relied on an analog circuit, which necessitated substantial circuit parameter modulation before detection. Furthermore, removing all impacts, particularly amplitude changes throughout time, may be difficult. For simplicity, we propose our demodulation method using digital technology.

### 3.1. Improved DC Component Elimination Algorithm

As the analysis above, the output amplitude fluctuates over time. However, because the partial discharge detecting frequency spectrum is broad (20–500 kHz), we only focus on the signals between 60 and 300 kHz. Two FRMs eliminate the amplitude shift induced by polarization fading, and the system acquisition time is adjusted to 100 ms using an oscilloscope with a 2 MHz sampling rate. These operations significantly suppress the *D_i_*(*t*) and *A_i_*(*t*) impacts. As a result, they can be viewed as a constant within a short data sampling time. In Arm#2, a phase modulator guarantees that every coupler output reaches its maximum value within the sampling time, allowing the amplitude to be correctly measured and the amplitude fluctuation to be overcome. Any element bearing a tunable vibration, such as piezoelectric ceramic, can be used in the phase modulator. To prevent overlapping with the partial discharge detecting frequency and to save cost, we use a commonly used modal shaker with 500 Hz 20 V in our system. The PD signal will be coupled to the output signal in a carrier wave due to the phase modulator’s introduction. The amplitude variation will not impact on the output maximum and minimum values throughout the sampling time. Following the above processes, the three-way output signals are represented as:

(3) $$U_{i}=D_{i}+A_{i}\cos[C\cos(1000\pi t)+\Phi (t)+\sigma (t)-(i-1)(\frac{2\pi }{3}+\theta_{i})]$$

where *C* is the phase modulator modulation coefficient, the output amplitude is readily obtained through Equation (3). Unfortunately, fluctuations in the output maximum and minimum values due to photodetector gain coefficient fluctuations are also conceivable. As a result, the duration of data required to compute the output amplitude must be rigorously regulated with one modulation cycle (2 ms), and the phase modulator must remain operational during the detection process. It implies that the INPS only concentrates on every 2 ms of data to ensure accuracy and then combines all findings after complete PD detection. Furthermore, if the amplitude fluctuation is extreme, the modulation frequency can be adjusted accordingly but not to the PD detection frequency. Compared to [21], this method will assure that amplitude parameters perform in real-time. The output amplitude in 2 ms of each path is:

(4) $$D_{i}=\frac{\max(U_{i})+\min(U_{i})}{2}\;A_{i}=\frac{\max(U_{i})-\min(U_{i})}{2}$$

The DC component is effectively eliminated by subtracting the DC amplitude from Equation (3).

### 3.2. Improved AGC Algorithm

The output eliminates the DC component after the improved DC component elimination procedure. However, the AGC algorithm cannot compute the AC amplitude accurately due to the asymmetric coupler. The last step (AGC) in the original NPS method has been pushed forward the DCM to avoid magnifying the amplitude change impact in following DCM algorithms, and the signal without DC amplitude is then divided by the AC amplitude of each output to achieve normalized results:

(5) $$U_{i}=\cos[C\cos(1000\pi t)+\Phi (t)+\sigma (t)-(i-1)(\frac{2\pi }{3}+\theta_{i})]$$

### 3.3. Improved DCM Algorithm

After the first two procedures, the output amplitude is removed. However, the *σ*(*t*) and the phase modulator introduced optical phase are still in (4). Fortunately, the above phases do not coincide with the partial discharge detecting frequency spectrum. Before the DCM process, 60–300 kHz band-pass digital filtering is applied. In the filtering process, the phase modulator introduced multiple frequency terms in the expansion of the Bessel function [17] located in 60–300 kHz

(6) $$2\sum_{k=60}^{300}{\left(-1\right)}^{k}J_{2k}(C)\cos2k(1000\pi t)$$

(7) $$2\sum_{k=60}^{299}{\left(-1\right)}^{k}J_{2k+1}(C)\cos(2k+1)(1000\pi t)$$

will be retained. Where *J*_2*k*_(*C*) *J*_2*k+*1_(*C*) are the 2*k*th and (2*k*+1)th order coefficient of the Bessel function. However, the modulation coefficient in our system is about 3, and *J*_120_(3)–*J*_600_(3) are too small for *J*_0_(*C*). Therefore, the results of (6) and (7) can be regarded as 0. The identical approximation is also used to handle the multiple frequency terms of *σ*(*t*). Then, the results containing only the measured phase are obtained:

(8) $$U_{i}=J_{0}(C)J_{0}(B)\cos[\Phi (t)-(i-1)(\frac{2\pi }{3}+\theta_{i})]$$

where *J*_0_(*C*), *J*_0_(*B*) are the zeroth order coefficient of the Bessel function, *C* and *B* are defined in the previous formula.

After the DCM algorithm for Equation (8), the result contains the derivative of Φ(*t*) is obtained:

(9) N=kΦ′(t)

where

(10) $$k=-\frac{J_{0}^{2}(C)J_{0}^{2}(B)}{2}\left\{\begin{array}{l} \sqrt{3}[\cos\theta_{0}+\cos\theta_{2}+\cos(\theta_{0}+\theta_{2})]\\ -\sin\theta_{0}-\sin\theta_{2}+\sin(\theta_{0}+\theta_{2})) \end{array}\right\}$$

is a constant coefficient introduced by the *θ_i_* and Bessel coefficient in Equation (8). Because *k* is a constant, the proportional result of Φ(*t*) can be obtained by integrating *N*:

(11) ∫Ndt=kΦ(t)

**Figure 2:**
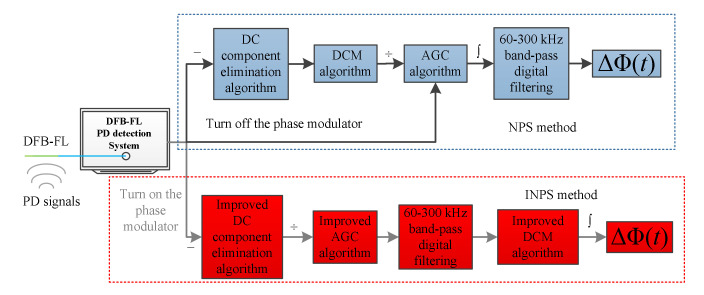
The flow chart of NPS and INPS. (The upper section shows the flow chart of NPS, the bottom shows the flow chart of INPS).

PD acoustic emission detection aims mainly to verify the PD occurrence. The result of Equation (11) is sufficient for this purpose. If a more accurate result is required in some conditions, the coupler phase estimation method in [26] can be used for Equation (11). The NPS and INPS flow charts are shown in Figure 2 to compare the differences in the calculating processes visually.

## 4. Experimental Results

**Figure 3:**
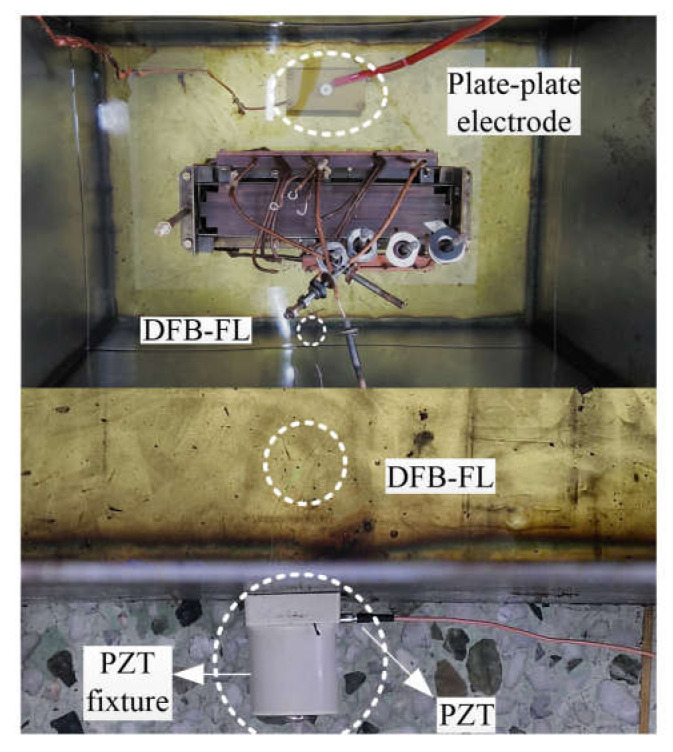
Experimental platform and install location of sensors. (The upper section shows the vertical view of the oil tank, and the bottom shows the installation distance between DFB-FL and PZT).

The experimental platform is depicted in Figure 3. The PD of oil-immersed transformers occurs mainly within the transformer windings, where the acoustic impedance mismatch caused by the liquid and solid causes ultrasonic attenuation. To imitate the interior environment of a genuine transformer, we installed an 80 kVA oil-immersed transformer winding in an iron tank. The plate–plate electrode and DFB-FL are submerged in oil and separated by the winding to investigate the INPS performance in the condition of significant ultrasonic attenuation. Our studies use a piezoelectric transducer sensor (PZT) (SR-150M) as a contrast sensor, which is mounted on the oil tank wall using a magnetic fixture and a 40 dB pre-amplifier to amplify the ultrasonic signal.

A thick layer of ultrasound coupling agent is applied to PZT’s surface to better couple the pressure waves. The distance between the PZT and DFB-FL is less than 5 cm. The high voltage is provided by a non-PD transformer, which steadily climbs in increments of 1 kV and maintains each voltage for 1 min. An oscilloscope (KEYSIGHT-DSOX 1204A) with a rising edge trigger mode records the PD signals detected by the system and PZT. The partial discharge analysis instrument (TCD-9302), the widely used device to judge the occurrence of PD by detecting the PD pulse, records the corresponding discharge magnitude. The computer completes the calculation of the INPS.

**Figure 4:**
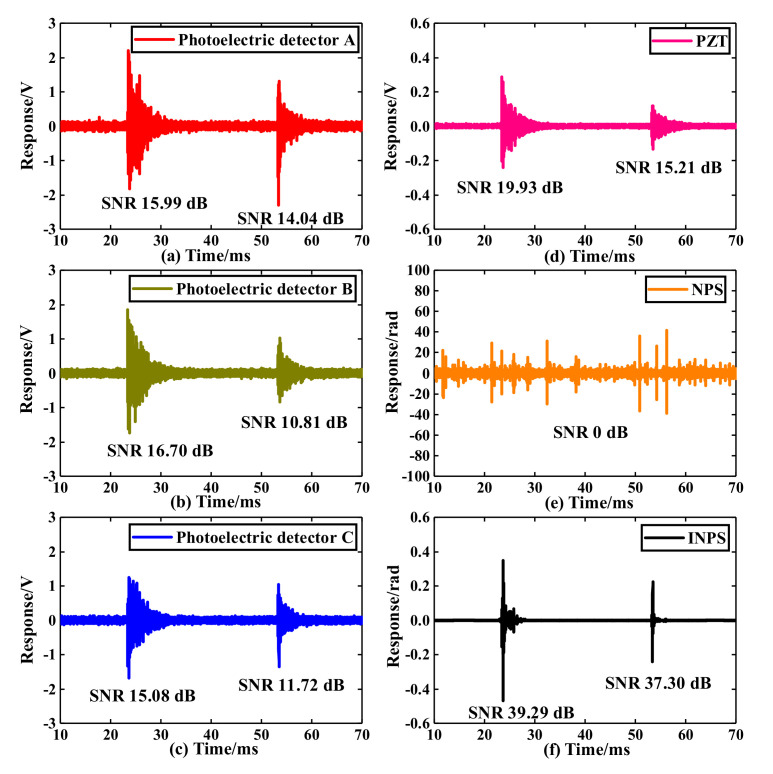
Results of PD detection in the liquid–solid composite dielectric ((**a**–**c**) are the results of three photodetectors with 60–300 kHz digital band-pass filtering, respectively. (**d**) is the result of PZT with the same band-pass filtering. (**e**,**f**) are the NPS and INPS method results, respectively.).

When the voltage is raised to 17.6 kV, the PZT and system simultaneously collect the PD signals. The average discharge magnitude observed by the PD analysis instrument in the 60 s measured 72.6 pC. The findings of the PD detection are shown in Figure 4.

Figure 4a–c display the results read from three photodetectors using 60–300 kHz digital filtering without further calculation, and they represent the ID method. Figure 4d shows the PZT result with the same digital filtering. Figure 4e shows the NPS result derived from Figure 4a–c. As can be seen from the NPS result, the asymmetric coupler dramatically limits the performance of NPS. Moreover, the *D_i_*(*t*) and *A_i_*(*t*) are functions of time that cannot be treated as a constant, introducing more variables in the NPS and amplifying the influence of coupler asymmetry. Finally, the NPS result was seriously ineffective and completely distorted. The specific influence of the asymmetric coupler can be referred to in [19]. The INPS method results are shown in (f). The result of the method is the phase information in the original signal. Compared to the ID method, the method efficiently eliminates the influences above, particularly the *D_i_*(*t*) and *A_i_*(*t*) influences. Therefore, the damping of the proposed demodulation method is different from the original signal. To quantitatively compare the performance of different methods, we define SNR = 10log10(*P_S_*/*P_N_*). Where *P_S_* represents the signal power, *P_N_* represents the noise power. The power is calculated by the following:

(12) $$P=\frac{1}{n}\sum_{k=1}^{n}S_{k}^{2}$$

where *S_k_* is the discrete data point in signal or noise, the three photodetectors’ average maximum peak-to-peak response value is about 3.51 V, measuring approximately 6.69 times greater than PZT. However, the average SNR of photodetectors A B C is 15.02 dB, 13.76 dB, and 13.4 dB, respectively. They are all lower than PZT. Compared to the results above, the average SNR of the proposed method is 38.30 dB, much higher than the ID method and the PZT result. The results prove the effectiveness of the proposed method for optical fiber PD detection sensors and show that the average SNR of the system is significantly increased by 24.2 dB when the amplitude noise in the system is thoroughly eliminated. The sensitivity advantage of DFB-FL is effectively brought into play, and the PD detection system’s ability to distinguish weak PD signals from the original background noise can be remarkably improved upon by the INPS method.

Combining all findings above, the INPS method exhibits an ultra-high demodulation SNR when compared to frequently used PD signal processing methods such as wavelet packet decomposition [27], passive homodyne [5,10], active homodyne [8,13], and quadrature interrogating method. Furthermore, the INPS demodulation system is substantially less expensive than the same system that uses an electro-optic modulator [2]. As a result, the INPS method can help develop any optical fiber sensors using the Michelson fiber interferometer as a demodulation system for PD detection.

## 5. Conclusions

In conclusion, a DFB-FL-based PD detection system was presented. By removing the direct impacts of the NPS method, an ultra-high SNR improved NPS (INPS) scheme for optical fiber sensors used in PD detection was proposed. The PD detection experiment in a liquid–solid composite dielectric was completed. The results of the experiments demonstrated that the system could identify PD signals in a large acoustic impedance environment. The INPS’s average SNR is 38.30 dB, measuring much greater than the ID method and PZT. With the INPS scheme, the system’s average SNR improved by 24.2 dB. Furthermore, the INPS avoids the challenges associated with 3 × 3 coupler asymmetry and is helpful for any optical fiber sensor to construct a detection system using a cheaper asymmetric 3 × 3 coupler when the detection frequency band is known.

## Data Availability

Data sharing is not available for this article.
